# IL-6 Receptor Is a Possible Target against Growth of Metastasized Lung Tumor Cells in the Brain

**DOI:** 10.3390/ijms14010515

**Published:** 2012-12-27

**Authors:** Mami Noda, Yukiko Yamakawa, Naoya Matsunaga, Satoko Naoe, Taishi Jodoi, Megumi Yamafuji, Nozomi Akimoto, Norihiro Teramoto, Kyota Fujita, Shigehiro Ohdo, Haruo Iguchi

**Affiliations:** 1 Laboratory of Pathophysiology, Graduate School of Pharmaceutical Sciences, Kyushu University, 3-1-1 Maidashi, Higashi-ku, Fukuoka 812-8582, Japan; E-Mails: ya0ka8@yahoo.co.jp (Y.Y.); ps107034@yahoo.co.jp (S.N.); mkmkblanket@yahoo.co.jp (T.J.); megumi-y@huk.bbiq.jp (M.Y.); mocchi-mochi-croissant-oomori@hotmail.co.jp (N.A.), fkyota@yahoo.co.jp (K.F.); 2 Department of Pharmaceutics, Graduate School of Pharmaceutical Sciences, Kyushu University, 3-1-1 Maidashi, Higashi-ku, Fukuoka 812-8582, Japan; E-Mails: matunaga@phar.kyushu-u.ac.jp (N.M.); ohdo@phar.kyushu-u.ac.jp (S.O.); 3 Department of Pathology, National Hospital Organization Shikoku Cancer Center, Matsuyama, Ehime 791-0280, Japan; E-Mail: nteramot@shikoku-cc.go.jp; 4 Department of Internal Medicine, National Hospital Organization Shikoku Cancer Center, Matsuyama, Ehime 791-0280, Japan; E-Mail: higuchi@shikoku-cc.go.jp

**Keywords:** brain metastasis, tumor microenvironment, lung cancer, HARA-B cells, anti-IL-6R antibody, astrocytes

## Abstract

In the animal model of brain metastasis using human lung squamous cell carcinoma-derived cells (HARA-B) inoculated into the left ventricle of the heart of nude mice, metastasized tumor cells and brain resident cells interact with each other. Among them, tumor cells and astrocytes have been reported to stimulate each other, releasing soluble factors from both sides, subsequently promoting tumor growth significantly. Among the receptors for soluble factors released from astrocytes, only IL-6 receptor (IL-6R) on tumor cells was up-regulated during the activation with astrocytes. Application of monoclonal antibody against human IL-6R (tocilizumab) to the activated HARA-B cells, the growth of HARA-B cells stimulated by the conditioned medium of HARA-B/astrocytes was significantly inhibited. Injecting tocilizumab to animal models of brain metastasis starting at three weeks of inoculation of HARA-B cells, two times a week for three weeks, significantly inhibited the size of the metastasized tumor foci. The up-regulated expression of IL-6R on metastasized lung tumor cells was also observed in the tissue from postmortem patients. These results suggest that IL-6R on metastasized lung tumor cells would be a therapeutic target to inhibit the growth of the metastasized lung tumor cells in the brain.

## 1. Introduction

In the metastatic process of tumor cells, the microenvironment of the metastatic site plays an important role in the tumor cells invasion and proliferation in the target tissues [1,2]. Such a microenvironment contains many resident cell types, in addition to metastatic tumor cells. In the brain or central nervous system (CNS), the microenvironment is composed of neurons, glial cells (microglia, astrocytes and oligodendrocytes) and blood vessels/pericytes. They are likely to interact with each other and stimulate each other, changing the characteristics and morphology.

In the CNS, activated glial cells contribute to the innate immune response and produce a large variety of different inflammatory mediators as a chronic inflammatory reaction [3]. A similar mechanism could function in mediating tumor cell survival, proliferation and colonization and invasion and motility in the microenvironment of brain metastases [4,5]. The involvement of brain-resident and infiltrating cells in the pathology of primary and metastatic brain tumors is gradually getting unveiled. A better understanding of the tumor microenvironment in the brain and interactions between each cell type is important for therapeutic treatment or even preventing metastases [6].

Experimental models of brain metastasis will aid as a microenvironment to support metastatic growth. In brain metastasis mouse models using human lung cancer cell lines, tumor cells metastasized to whole regions of the brain. It is important to understand the interactions between invaded tumor cells and resident brain cells to understand how tumor cells grow and rapidly colonize the brain. Similar results were seen with breast cancer brain metastasis models [5,7]. Further, this understanding could help prevent the growth of metastatic tumors in the brain.

Though the interaction between each cell types is complicated, identification of the factors interacting between two cell types is possible *in vitro*, using primary cultured cells. Molecules, most likely cytokines and growth factors, released by interaction of these two cell types may also influence other cell types.

Among the glial cells in the brain, astrocytes are the most abundant cell population and play an important role in maintaining homeostasis of the brain. Astrocytes have been shown to produce a wide variety of cytokines and growth factors; among them, it was suggested that IL-6, TNF-α and IL-1β may contribute to the development of brain metastasis by lung cancer cells [8], and IL-6, TGF-β and IGF-1 by breast cancer cells [9]. Interestingly, receptor expression for IL-6 increased with time, while receptor expression for TNF-α and IL-1β decreased with time, in the case of lung cancer cells interacting with astrocytes [8]. Therefore, in the present study, we tested the effects of the inhibition of the IL-6 receptor on tumor cells to see if tumor cell growth would be inhibited and if it could be the therapeutic target.

## 2. Results and Discussion

### 2.1. Increase of IL-6 Receptor in Lung Cancer Cells *in Vitro* and *in Vivo*

**Figure 1:**
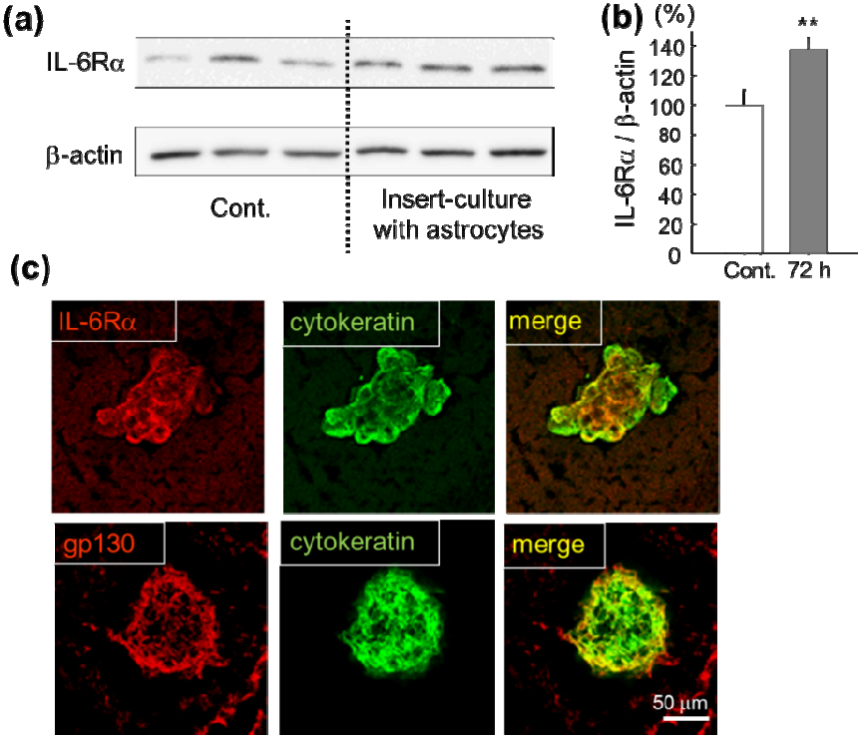
Increased expression of IL-6 receptor (IL-6Rα) and receptor subunit (gp130) in lung tumor cells. (**a**) IL-6Rα protein in cultured human lung cancer cell line (HARA-B cells) was detected by Western blot analysis of IL-6Rα protein using its specific antibody. Three cases with or without (Cont.) stimulation of HARA-B cells by insert-cultured astrocytes for 72 h are shown; (**b**) Histograms of the relative band density ratio of total IL-6Rα protein normalized to the levels of β-actin. ** *p* < 0.01 compared with unstimulated control HARA-B cells; (**c**) Immunohistochemistry of IL-6Rα and gp130 with HARA-B cells (cytokeratin) in brain slices from nude mice at four-five weeks after inoculation of HARA-B cells into left ventricle of the heart.

The increased expression of IL-6 receptor (IL-6Rα) in human lung squamous cell carcinoma-derived cells (HARA-B) has been shown immunocytochemically under co-culture with primary cultured mouse astrocytes [8]. To confirm the increase of IL-6Rα quantitatively, the protein level of IL-6Rα in HARA-B cells was tested by Western blotting after culturing with or without mouse astrocytes for 72 h using insert-culture to prevent physical contact (Figure 1a). The quantitative analyses showed a significant increase in IL-6Rα after 72 h when HARA-B cells were insert-cultured with astrocytes (Figure 1b).

Not only the *in vitro* conditions, but strong expression of the IL-6 receptor and receptor subunit (IL-6Rα and gp130) on HARA-B cells were observed in the brain slices obtained from metastasized brain at four-five weeks after inoculation of HARA-B cells into the left ventricle of the heart in nude mice (Figure 1c).

### 2.2. Effect of IL-6 Receptor Antibody on Lung Cancer Cells

#### 2.2.1. Effect of Monoclonal Antibody against Human IL-6 Receptor (Tocilizumab) on the Growth of HARA-B Cells *in Vitro*

**Figure 2:**
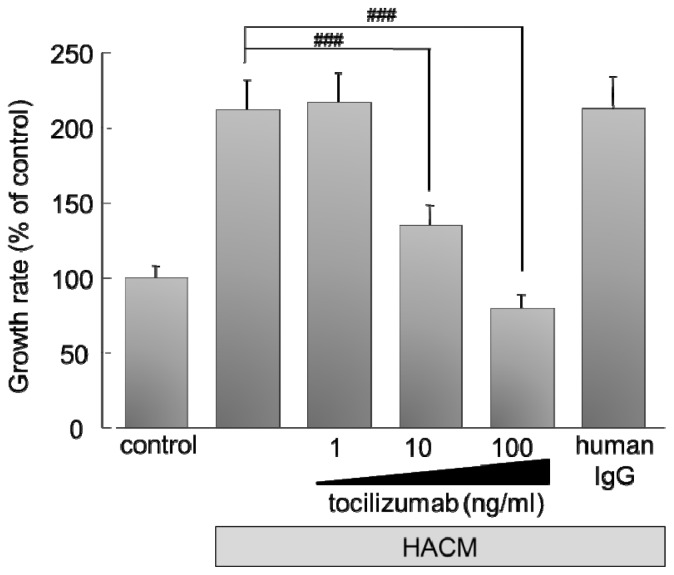
Stimulating effect of astrocytes on tumor cell proliferation and inhibitory effects of monoclonal antibody against human IL-6R (tocilizumab) on stimulated tumor cell proliferation. Proliferation of HARA-B cells was significantly increased after being cultured with HARA-B-stimulated astrocyte conditioned medium (HACM). Data were given as the percentage of tumor cell proliferation without HACM. HARA-B cells were cultured for 24 h in DMEM, then in serum-free DMEM for 24 h and then for 48 h in HACM (serum-free), with or without tocilizumab (1, 10, and 100 ng/mL) or human IgG as the negative control. Control cells were cultured with serum-free DMEM instead of HACM. Each value represents the mean ± SEM (*n* = 19).

To inhibit the IL-6R expressed on human-derived HARA-B cells, we used humanized anti-human IL-6 receptor (IL-6R) antibody, tocilizumab, which is used for treatment in autoimmune diseases [10,11], such as rheumatic disease [12–19], Crohn’s disease [20] or Castleman disease [21,22]. Proliferation of HARA-B cells was increased by soluble factors, such as IL-1β, TNF-α and IL-6, released by HARA-B-stimulated astrocytes, as reported previously [8]. HARA-B-stimulated astrocyte conditioned medium (HACM), therefore, increased HARA-B cell proliferation significantly. However, adding tocilizumab to HACM inhibited the cell proliferation (growth rate) of HARA-B cells in a dose-dependent manner (Figure 2). The increased growth rate by HACM was reduced almost completely by 100 ng/mL tocilizumab, suggesting that IL-6 would play the main role in HACM, rather than IL-1β and TNF-α.

#### 2.2.2. Effect of Tocilizumab on the Brain Metastasis of HARA-B Cells *in Vivo*

**Figure 3:**
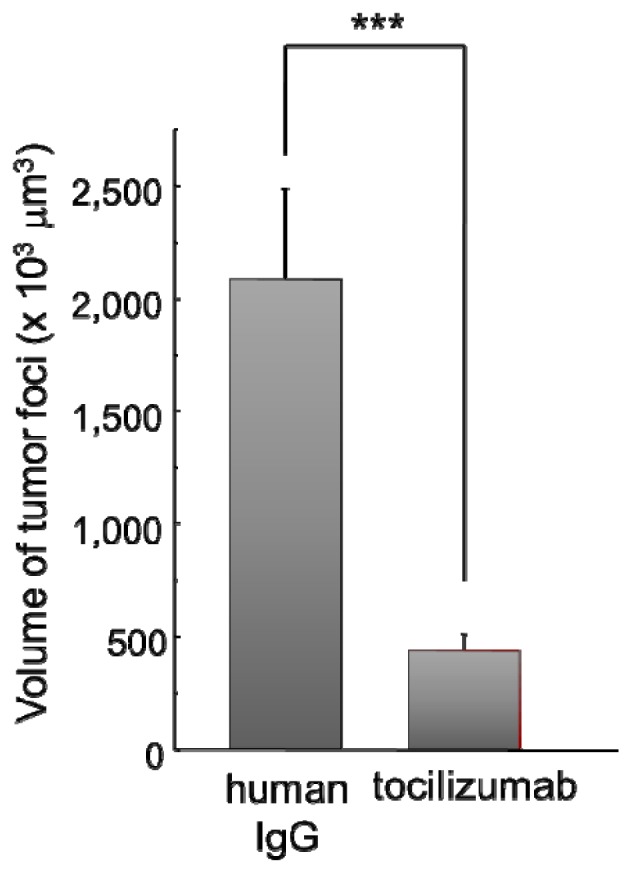
Effects of tocilizumab on lung tumor metastases to the brain. Intravenous injection of tocilizumab (1.0 mg/100 μL) twice a week for three weeks (six injections) inhibited the size of metastatic tumor foci significantly. Three dimensional volume of tumor foci in 30 μm brain slices from the whole brain, except the olfactory bulb and the cerebellum, was calculated (control; *n* = 254, tocilizumab; *n* = 549). *** *p* < 0.005.

Next, we tested the effects of tocilizumab in a brain metastasis model of lung tumor cells by inoculating HARA-B cells into the left ventricle of the heart of nude mice [8]. After three weeks of inoculation of HARA-B cells, being the time for tumor cells to start metastasizing to the brain, intravenous injection of tocilizumab was given twice a week for three weeks. Then, the size of each focus was investigated. The volume of each metastatic focus was significantly smaller in tocilizumab-injected mouse brains (Figure 3). The incidence of tumor metastases was different in each animal in both human IgG- and tocilizumab-injected mouse brains. The body weight of each mouse was similar in both the human IgG- and tocilizumab-injected group (not shown). Whether earlier injection of tocilizumab could decrease the incidence of brain metastasis remains to be elucidated.

### 2.3. Expression of IL-6R in Postmortem Human Brain

**Figure 4:**
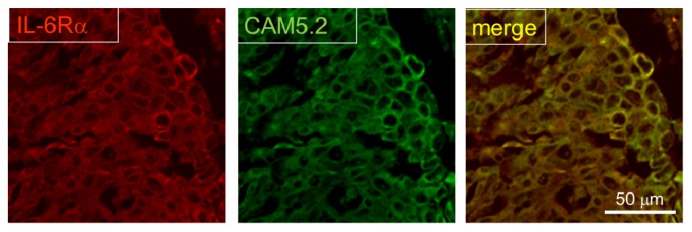
Immunostaining of IL-6R and tumor cells from human brain section. IL-6R-positive cells are merged with aggregated cancer cells (CAM5.2).

Finally, we checked the expression of IL-6R in postmortem human brain from lung cancer patients. It was shown that astrocytes accumulate around metastasized lung cancer cells in human brain [8]. At metastasized foci, IL-6R was strongly stained (Figure 4), suggesting that up-regulation of IL-6R also occurs in the brain metastases of lung cancer in human.

In our previous study [8], three other cell lines derived from human squamous cell carcinoma (QG56, EBC-1) and non-small cell lung cancer (PC-9) were examined *in vitro*, showing the similar responses to the medium from insert-culture with astrocytes. Those results suggest that mutual activation of astrocytes and lung tumor cells are common phenomena and IL-6 plays an important role in the progression of tumor cells in the microenvironment of the metastasized brain area.

Though brain metastases, especially with lung cancer, melanoma and breast cancer, are becoming a common clinical problem, limited treatment options have existed [23], such as gamma knife radiosurgeries [24,25], stereotactic radiosurgery [26] or irradiation [27], UVC irradiation [28] and whole brain radiotherapy [29–31]. Recently, a potential role of targeted agents, including antiangiogenic compounds and epithelial growth factor receptor inhibitors, have been implicated for the prevention of brain metastasis formation in breast cancer or non-small cell lung cancer [32]. MicroRNAs [33,34], targeting angiogenesis [35] and cytotoxic chemotherapy, in some specific situations [36], might be potential options. In addition to them, our findings may also contribute to a better control or inhibition of metastatic lung tumor growth in the brain.

## 3. Experimental Section

### 3.1. Cell Culture

Primary glial cell cultures were performed according to the method described previously [37,38]. Briefly, the cerebral corteces obtained from one-day-old C57BL/6 mice (Kyudo, Kumamoto, Japan) were isolated under a dissecting microscope and carefully separated from the choroid plexus and meninges. The isolated cerebral corteces were minced and treated with trypsin-EDTA solution (0.25% trypsin, 1 mM EDTA) and 1500 U DNase in Dulbecco’s modified Eagle medium (DMEM; Nissui, Tokyo, Japan) at 37 °C for 10 min. Cell suspensions were filtered through 70 μm pore size mesh (BD Falcon, Bedford, MA, USA) into DMEM containing 10% fetal calf serum (FCS; Hyclone, UT, USA), 2 mM l-glutamine, 100 U/mL penicillin, 100 μg/mL streptomycin, 0.37% NaHCO_3_ and 110 μg/mL pyruvic acid. After centrifugation, cells were filtered through 40 μm pore size-mesh (BD Falcon), plated into a poly-l-lysine coated 75 cm^2^ cell culture flask at the density of two brains per flask in 10 mL of DMEM and maintained at 37 °C in 10% CO_2_–90% air, with a change of the medium twice per week. Astrocytes were obtained after 28 days of mixed glial cell cultures as follows. After removing other glial cells by shaking the flasks, the astroglial layer was removed from the flasks by treatment with trypsin-EDTA solution (0.06% trypsin, 0.25 mM EDTA in serum free DMEM) at 37 °C for 45 min. Suspended astrocytes were filtered through 40 μm pore size-mesh and seeded. Astrocyte purity ranged from 90% to 95%, as determined by immunostaining with anti-GFAP antibody (Sigma, St. Louis, MO, USA) (data not shown). Astrocytes were maintained in the same medium used for cell suspension from the cerebral cortex at 37 °C in 10% CO_2_–90% air. HARA-B cells were maintained under the same condition. Cells were grown in a 25 cm^2^ cell culture flask (Nalge Nunc International, Napersville, IL, USA), and single-cell suspensions of the cells were obtained by trypsin treatment.

### 3.2. Western Blotting

The expression protein level of IL-6Rα in HARA-B cells was examined by Western blotting relative to β-actin. Though β-actin is frequently too abundant to serve as a suitable control for lower-abundance proteins, the signal was not saturated nor near-saturated in our case.

HARA-B cells cultured with or without insert-culture of astrocytes were collected and homogenized in a lysis buffer containing protease inhibitor cocktail (Sigma, St. Louis, MO, USA, 2%) and phosphatase inhibitor cocktail (Sigma, St. Louis, MO, USA, 1%). Twenty mg of proteins were loaded for each lane, separated by SDS-PAGE gel (10%) and transferred to PVDF membrane (Bio-Rad, Hercules, CA, USA). The membrane was blocked with 5% low-fat dried milk and incubated with the following for 1 h at room temperature: rabbit polyclonal anti-human IL-6Rα antibody (Santa Cruz, CA, USA) (1:100) and mouse monoclonal antibody anti-β-actin (1:200, Sigma, St. Louis, MO, USA). The membrane was washed and incubated with specific secondary antibodies (Amersham ECL anti-rabbit IgG and anti-mouse IgG horseradish peroxidase-linked species-specific whole antibody, 1:1000, GE Healthcare, Piscataway, NJ, USA). The blots were detected by use of an ECL Western blotting detection system (GE Healthcare) and LAS-4000 imaging system (Fujifilm, Tokyo, Japan). Bands were quantified using the software Multi Gauge (Fujifilm).

### 3.3. Experimental Model for Brain Metastasis

The study was approved by the Animal Care and Use Committee at Kyushu University and carried out in accordance with the National Institutes of Health Guide for the Care and Use of Laboratory Animals. Male five-week-old nude mice (BALB/c *nu*/*nu*) (Kyudo, Kumamoto, Japan) kept in a specific pathogen-free environment were used. A single suspension of human lung squamous cell carcinoma-derived cells (HARA-B) (2 × 10^5^ cells/0.2 mL PBS) was inoculated into the left ventricle of the heart in nude mice according to the method described previously.

### 3.4. Human Tissue Samples

A total of 6 paraffin-embedded samples from patients with lung tumor brain metastasis were used. All sections were obtained from the National Hospital Organization Shikoku Cancer Center. Use of the human specimens was in accordance with the University Ethics Commission. The formalin-fixed, paraffin-embedded archival tissue blocks were retrieved, and matching hematoxylin and eosin (H & E)-stained slides were reviewed and screened for representative tumor regions by a neuropathologist.

### 3.5. Immunohistochemistry

Nude mice were perfused transcardially with 50 mL of 10 U/mL heparin and 0.5% procaine in PBS and 4% paraformaldehyde (PFA) in PBS prior to excision of the brain. Then, the brain was removed, post-fixed for 3 h and cryoprotected for 24 h in PBS containing 20% sucrose. The brain was cut into slices (30 μm thick) using a cryostat, and the sections were placed on glass slides. In order to acquire the better immunoreactive images, sections were autoclaved with 0.01 M citrate buffer solution (pH 6.0), permeabilized with 0.3% TritonX-100 in PBS for 15 min and then blocked in BlockAce (Dainippon Pharmaceutical, Japan) for 1 h at room temperature. Sections were incubated with mouse anti-human cytokeratin monoclonal antibody (AE1/AE3 pool of cytokeratin) (Dako, Glostrup, Denmark, 1:100) at 4 °C overnight. Biotinylated anti-mouse IgG (Jackson, 1:200) were incubated for 2 h at room temperature, followed by the incubation with streptavidin Alexa488 (Molecular Probes, 1:500) for 2 h at room temperature. For double-staining of cytokeratin and IL-6R or gp130, sections were incubated with rabbit polyclonal anti-human IL-6Rα antibody (Santa Cruz, CA, USA) (1:200), rabbit anti-human gp130 antibody (Santa Cruz, 1:500) at 4 °C overnight after staining of cytokeratin. The cells were then incubated for 5 h at room temperature with secondary antibody, containing FITC-conjugated anti-mouse IgG (Sigma, St. Louis, MO, US, 1:500) and Cy3-conjugated anti-rabbit IgG (Jackson ImmunoResearch Laboratories, Inc., West Grove, PA, USA, 1:500). Every treatment was followed by washing three times with PBS containing 0.3% TritonX-100 for 5 min.

The sections were mounted in the Perma Fluor Aqueous Mounting Medium (Thermo Shandon, Pittsburgh, PA, USA) and analyzed with a confocal microscope (LSM510 META, Carl Zeiss, Co. Ltd. Germany). Z-stack images were obtained from each section by LSM 510 META and total intensity was calculated by a LSM image browser.

As for human tissue samples, after removal of paraffin in xylene and rehydration in a grade of alcohols (100%, 90%, 80%, 70% and 60%), sections were incubated for 30 min in 0.05 M phosphate buffer pH 7.6 containing tripsin and KCl for antigen retrieval. Then, the sections were incubated for 1 h in 0.3% H_2_O_2_, and blocked in PBS containing 1% BSA and 5% normal donkey serum (Jackson Immuno Research Laboratories Inc., West Grove, PA, USA) for 1 h at room temperature. Then, the sections were incubated with anti-human IL-6Rα antibody (1:200) at 4 °C overnight, Cy3-conjugated anti-rabbit IgG (Jackson ImmunoResearch Laboratories, Inc., West Grove, PA, USA, 1:500) for 3 h at room temperature and FITC-conjugated anti-human cytokeratin antibody (CAM5.2) (Becton-Dickinson Biosciences, New Jersey, USA) (undiluted solution) for 1 h at room temperature. Every treatment was followed by washing three times with PBS containing 0.3% TritonX-100 for 5 min. The sections were mounted in the Perma Fluor Aqueous Mounting Medium (Thermo Shandon, Pittsburgh, PA, USA) and analyzed with a confocal microscope (LSM510 META, Carl Zeiss, Co. Ltd. Germany).

### 3.6. Cell Proliferation Assay

HARA-B-stimulated astrocyte-conditioned medium (H-ACM) was obtained as follows. The culture medium of HARA-B cells (5 × 10^3^ cells/well) was added to astrocytes cultures (5 × 10^4^ cells/well) and incubated for 24 h. Then, the medium was collected and centrifuged to remove debris (1500 rpm for 10 min at 4 °C) before use. For staining, cells were fixed with 4% PFA for 30 min at room temperature and permeabilized with 0.3% TritonX-100 in PBS for 15 min, followed with a blocking solution containing 1% BSA and 5% normal donkey serum (Jackson ImmunoResearch, West Grove, PA, USA) in PBS for 1 h at room temperature. Then, cells were incubated with mouse anti-human cytokeratin monoclonal antibody (AE1/AE3 pool of cytokeratin) (Dako, Glostrup, Denmark) (1:100) at 4 °C overnight, followed by incubation with the secondary antibody (FITC-conjugated anti-mouse IgG; Sigma, St. Louis, MO, USA, 1:500) for 5 h at room temperature and, then, incubated with 300 nM 4′,6′-diamidino-2-phenylindole hydrochloride (DAPI, Sigma, St. Louis, MO, USA) for 30 min at room temperature. The number of HARA-B cells in each well, which were positively stained with an anti-human cytokeratin antibody, was counted using a digital camera system (Axio Cam, Carl Zeiss) mounted on a light and fluorescent microscope (Axopscope2 plus, Carl Zeiss). The results were expressed as the percentage of control (single cell culture of HARA-B cells).

### 3.7. *In Vivo* Anti-Tumor Activity

After inoculation of HARA-B cells into the left ventricle of the heart of nude mice, the metastases of tumor cells were recognized at about three weeks. Therefore, after three weeks of inoculation, either tocilizumab (1.0 mg/100 μL) or human IgG (1.0 mg/100 μL) was injected intravenously twice a week during the following three weeks. The amount of tocilizumab was calculated from clinical dosage (8 mg/kg, every two weeks). Even though it has low permeability of the blood-brain barrier (1000–10,000 times lower concentration in the brain), it was estimated to be similar to the effective concentration *in vitro*. Then, the animals were anesthetized, perfused and fixed, as described above. The brain was cut into 30 μm in thickness using a cryostat. All sections were stained with DAPI (Sigma, St. Louis, MO, USA) for 10 min, then washed with PBS and coverslipped. The volume of tumor foci was calculated according to the following formula: tumor volume (mm^3^) = (major axis) × (minor axis)^2^ × 0.52 [39,40]. The major axis is the longest diameter of the tumor, and the minor axis is the maximal line drawn perpendicular to the major axis [41,42].

### 3.8. Statistical Analysis

All data are presented as mean ± SEM. One-way analysis of variance (ANOVA) and a *post-hoc* Bonferroni/Dunn test were used to examine the statistical differences. Differences were considered significant at *p* < 0.05.

## 4. Conclusions

In the animal model of brain metastasis using human lung squamous cell carcinoma-derived cells (HARA-B), the microenvironment of the metastasized tumor cells are important for tumor growth. Among the interaction between metastasized tumor cells and brain resident cells, tumor cells and astrocytes have been reported to stimulate each other, releasing soluble factors from both sides, subsequently promoting tumor growth significantly. Among soluble factors released from astrocytes, IL-6 was most likely responsible for tumor growth, because only the expression of IL-6R on tumor cells was up-regulated during the activation with astrocytes. Upon application of monoclonal antibody against human IL-6R (tocilizumab) to the activated HARA-B cells, the stimulated growth of HARA-B cells was significantly inhibited. When injecting tocilizumab to the animal model of brain metastasis at about the time when HARA-B cells start to metastasize to the brain, the growth of the foci was significantly inhibited. These results suggest that IL-6R on metastasized lung tumor cells would be a therapeutic target to inhibit at least the growth of the metastasized lung tumor cells in the brain.
